# Acylation, a Conductor of Ghrelin Function in Brain Health and Disease

**DOI:** 10.3389/fphys.2022.831641

**Published:** 2022-06-30

**Authors:** Alanna S. Thomas, Martina Sassi, Roberto Angelini, Alwena H. Morgan, Jeffrey S. Davies

**Affiliations:** Molecular Neurobiology, Institute of Life Sciences, School of Medicine, Swansea University, Swansea, United Kingdom

**Keywords:** acylation, acyl-ghrelin, unacyl-ghrelin, octanoic acid, ghrelin-O-acyl transferase, neurogenesis, neurodegeneration, beta-oxidation

## Abstract

Acyl-ghrelin (AG) is an orexigenic hormone that has a unique octanoyl modification on its third serine residue. It is often referred to as the “hunger hormone” due to its involvement in stimulating food intake and regulating energy homeostasis. The discovery of the enzyme ghrelin-O-acyltransferase (GOAT), which catalyses ghrelin acylation, provided further insights into the relevance of this lipidation process for the activation of the growth hormone secretagogue receptor (GHS-R) by acyl-ghrelin. Although acyl-ghrelin is predominantly linked with octanoic acid, a range of saturated fatty acids can also bind to ghrelin possibly leading to specific functions. Sources of ghrelin acylation include beta-oxidation of longer chain fatty acids, with contributions from fatty acid synthesis, the diet, and the microbiome. In addition, both acyl-ghrelin and unacyl-ghrelin (UAG) have feedback effects on lipid metabolism which in turn modulate their levels. Recently we showed that whilst acyl-ghrelin promotes adult hippocampal neurogenesis and enhances memory function, UAG inhibits these processes. As a result, we postulated that the circulating acyl-ghrelin:unacyl-ghrelin (AG:UAG) ratio might be an important regulator of neurogenesis and cognition. In this review, we discuss emerging evidence behind the relevance of ghrelin acylation in the context of brain physiology and pathology, as well as the current challenges of identifying the provenance of the acyl moiety.

## Introduction

Ghrelin is a stomach peptide often referred to as “the hunger hormone” due to its ability to stimulate the sensation of hunger during periods of low energy balance (Date et al., 2000; Nakazato et al., 2001; Kojima et al., 2009; Tamboli et al., 2017).

Within the CNS, ghrelin regulates brain plasticity involved in regulating memory and anxiety (Hornsby et al., 2020). Although its complex physiological effects have just begun to be characterized, ghrelin is now considered a critical factor in gut-brain signaling with relevance to neurodegenerative diseases, particularly dementia (Buntwal et al., 2019).

The activity of ghrelin is dependent on a specific post-translational step, whereby the peptide is acylated, mainly by octanoic acid, on the third amino acid, a serine residue to form acyl-ghrelin (Kojima et al., 1999). In the absence of acylation (the attachment of the fatty acid) ghrelin cannot bind and activate GHS-R. In this review, we focus on the biochemistry of acyl-ghrelin function.

Ghrelin is a 28 amino acid peptide, first identified in 1999 as the endogenous ligand for the growth hormone secretagogue receptor-1a (GHS-R1a) and is essential for growth hormone (GH) release. Acyl-ghrelin mediates the beneficial effects associated with calorie restriction (CR) in neurodegenerative disorders, including Parkinson’s Disease (PD) (Andrews et al., 2010). Indeed, pre-clinical models demonstrate that acyl-ghrelin promotes neurogenesis and enhances memory function (Walker et al., 2015; Hornsby et al., 2016; Kent et al., 2015; Menzies et al., 2014; Moon et al., 2014), whilst we have recently shown that UAG reduces neurogenesis and impairs memory function, with clinical data suggesting that the circulating AG: UAG ratio may be a valuable diagnostic biomarker of dementia (Hornsby et al., 2020). Thus, as the acylated form of ghrelin is essential for the function and activation of GHS-R1a—acyl-ghrelin is often considered to be the ‘active’ form of ghrelin. Of note, the Ser3 ghrelin residue, along with its acylation, are both conserved in vertebrates (Table 1) (Kojima and Kangawa, 2005). Mutation of this serine residue prevents the acylation of ghrelin by ghrelin-o-acyltransferase (GOAT) (Yang et al., 2008b), unless the substitution is for threonine (Darling et al., 2015), which is consistent with threonine being present at this position in certain amphibians (Kaiya et al., 2001).

**TABLE 1:** The amino acid sequence of ghrelin from a range of vertebrate species.

Mammalian	1*	10	20	28
Human	GSSFLSPEHQRVQQRKESKKPPAKLQPR			
Mouse/Rat	GSSFLSPEHQKAQQRKESKKPPAKLQPR			
Dog	GSSFLSPEHQKLQQRKESKKPPAKLQPR			
Rhesus monkey	GSSFLSPEHQRAQQRKESKKPPAKLQPR			
Bovine	GSSFLSPEHQKLQ - RKEAKKPSGRLKPR			
Sheep	GSSFLSPEHQKLQ - RKEPKKPSGRLKPR			
Avian	1*	10	20	26
Chicken	GSSFLSPTYKNIQQQKDTRKPTARLH			
Turkey	GSSFLSPAYKNIQQQKDTRKPTARLHPR			
Goose	GSSFLSPEFKKIQQQNDPAKATAKIH			
Fish	1*	10	20	23
Rainbow trout	GSSFLSPSQKPQVROGKGK-PPRV-amide			
Goldfish	GTSFLSPAQKPQ—GRRPPRM-amide			
Zebrafish	GTSFLSPTQKPQ—GRRPPRV-amide			

For each species, the third amino acid of the ghrelin peptide sequence is a serine residue and is the location for acyl-modification (indicated by asterisks). As shown, there is homology between the NH_2_-terminal sequences of ghrelin from these vertebrate species.

The acylation of ghrelin shares differences and similarities with other types of protein lipidation, the process through which a variety of lipids including cholesterol, isoprenoids and fatty acids are covalently attached to proteins during and/or after translation. These modifications including glycosylphosphatidylinositol (GPI) anchor, cholesterylation, N-myristoylation, palmitoylation, and prenylation regulate protein localization to membranes, protein interactions and function in signaling cascades. However, whilst lipidation was historically regarded as a relatively static modification providing membrane affinity or anchoring, the recent study of ghrelin and other proteins that are rapidly and reversibly acylated has rendered protein acylation a new signaling pattern relevant to many pathways. Although the acylation of ghrelin was the first demonstration of such a post-translational modification of a peptide, other important proteins such as the oncogenic regulator Wnt (Coombs et al., 2010; Herr and Basler, 2012; Janda et al., 2012; Willet et al., 2003; Nile and Hannoush, 2016; Takada et al., 2006), Hedgehog precursor proteins (Chamoun et al., 2001; Chen et al., 2004), and histocompatibility antigens (Schultz et al., 2018) can also be acylated by fatty acids that alter signaling capabilities. These alterations are modulated by the lipid actively participating in the mechanistic interactions that induce the conformational change required by the protein to exert its function. The linked lipid is therefore not a passive bystander but actively participates in the cascade of events. Interestingly, ghrelin, hedgehog and Wnt proteins are all acylated by members of the diverse family of membrane-bound O-acyl transferase (MBOAT) enzymes. Nonetheless, ghrelin is seemingly unique in its ability to bind fatty acids of varying lengths with higher specificity for octanoic acid. Studies into the physiological effects of post-translational acyl modifications are warranted.

**FIGURE 1:**
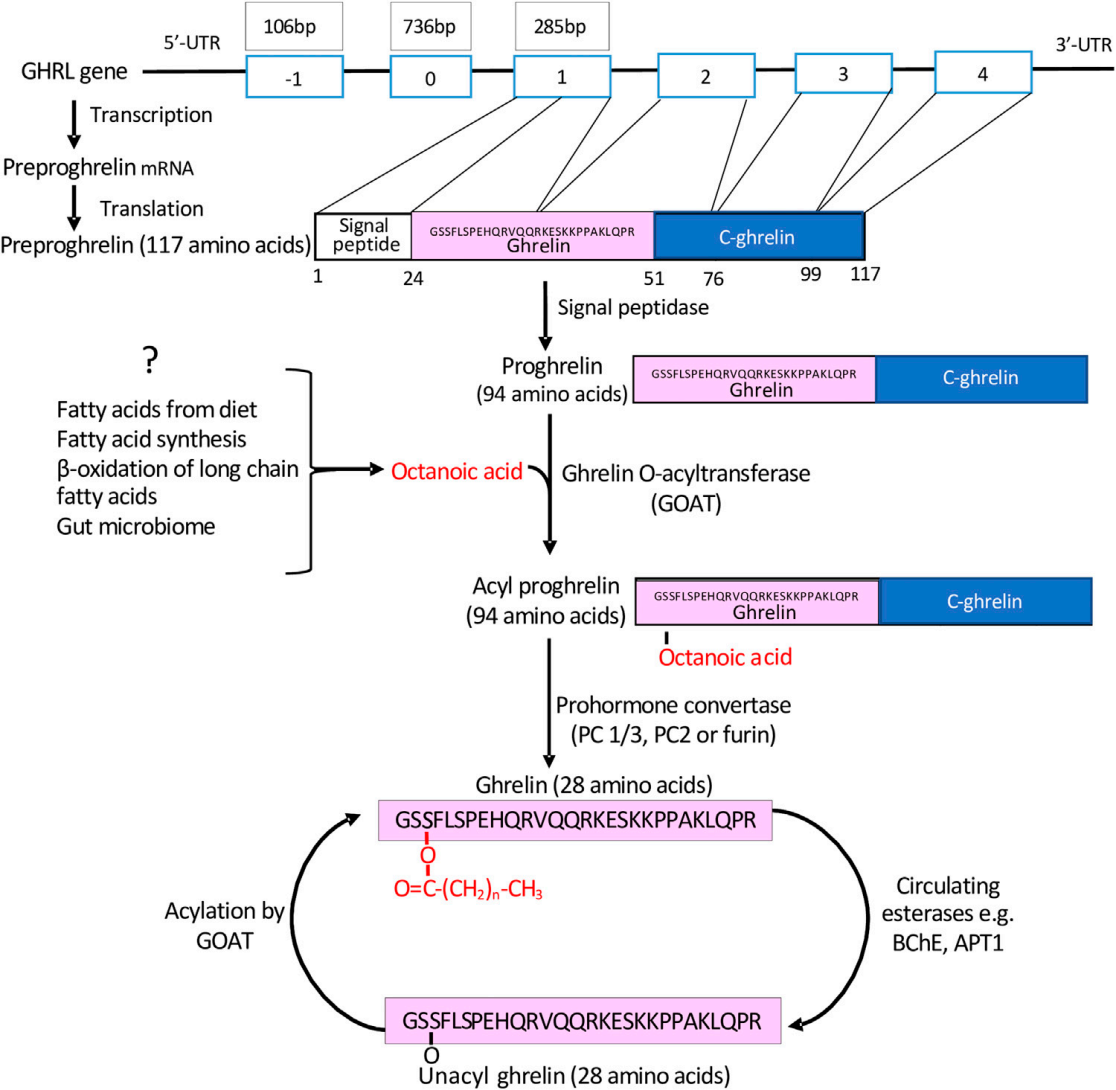
The generation of mature acyl-ghrelin. Following transcription and translation of the GHRL gene, a preproghrelin peptide of 117 amino acids is generated. This undergoes proteolytic cleavage to generate proghrelin (94 amino acids). Either proghrelin or mature ghrelin (28 amino acids) can undergo acylation by GOAT, which is predominately bound by C8. Acyl-ghrelin may be deacylated by esterases and then reacylated by GOAT at an alternative location.

The human ghrelin (GHRL) gene on chromosome 3p25-26 (Figure 1) encodes a 117-amino-acid precursor peptide, preproghrelin, which undergoes proteolytic cleavage in the endoplasmic reticulum (ER) to produce a 94 amino acid proghrelin peptide (Zhu et al., 2006). This is cleaved in the Golgi by pro-hormone convertase 1/3 (PC1/3) to generate the mature 28 amino acid ghrelin peptide (Liu et al., 2011). Notably, both proghrelin and ghrelin can be acylated by the enzyme GOAT (Zhu et al., 2006; Yang et al., 2008b).

In humans, intravenous acyl-ghrelin has a half-life of ∼9–13 min (Liu et al., 2011). Indeed, ∼90% of ghrelin detected in the circulation is UAG (Hosada et al., 2003), suggesting that its acylation is a rapid, reversible and tightly regulated process. Notably, within several cell types and tissues, ghrelin can be enzymatically acylated or de-acylated to permit fine cell-specific modulation of GHS-R1a signaling (Zeidman et al., 2009). Although several studies suggest that acyl-ghrelin and UAG may have distinct functions, with UAG no longer regarded as an inactive form of the peptide, it must be highlighted that for the rapid interconversion of UAG into acyl-ghrelin, basal levels of the peptide precursor are required, together with the necessary fatty acid.

## Acylation of Ghrelin by Ghrelin-O-Acyltransferase

In 2001, the human medullary thyroid carcinoma cell line (TT) was shown to generate UAG, enzymatically acylate UAG to become acyl-ghrelin, and secrete this into the media (Kanamoto et al., 2001). The subsequent generation of a ghrelin acylation cell culture system led to the identification of GOAT as the enzyme catalyst (Gutierrez et al., 2008). Also in 2008, mouse genome analysis identified membrane bound-O-acyl transferase (MBOAT4) as the gene (located on chromosome 8p12) that encodes for GOAT (Yang et al., 2008a). In agreement, acyl-ghrelin is not detected in GOAT null mice (Gutierrez et al., 2008) and the inhibition of GOAT leads to a decreased presence of acyl-ghrelin in insulinoma cells (Yang et al., 2008a), HEK and HeLa cells (Barnett et al., 2010) as well as in mouse serum (Yang et al., 2008a).

Other than GOAT, only two additional members of the MBOAT family, Porcupine (Porcn) and hedgehog acyl transferase (Hhat), are known to transfer fatty acids onto proteins. Other members transfer fatty acids to more complex lipids (Hofmann, 2000; Chang and Magee, 2009). GOAT is a membrane-bound enzyme-containing 11 transmembrane domains and is predominantly expressed in the stomach (Hofmann, 2000). The structure of GOAT is not fully resolved, and the enzyme’s active site and substrate binding site are still unknown (Taylor et al., 2013). However, it contains two highly conserved residues: asparagine (Asn307)—which seems to be essential for the interactions with the substrates, and histidine (His338)—thought to be involved in the catalytic mechanism (Taylor et al., 2013; Masumoto et al., 2015). Knowledge of GOAT’s ability to recognize ghrelin and fatty acyl-CoA substrates was obtained from biochemical and functional studies (Van Der Lely et al., 2004; Yang et al., 2008b; Darling et al., 2013; Darling et al., 2015) that suggest ghrelin as the unique substrate for GOAT (Darling et al., 2015). Generally, GOAT localization closely follows ghrelin expression, with high expression in the gastric oxyntic mucosa of rats and mice (Stengel et al., 2010), and humans (Gutierrez et al., 2008; Lim et al., 2011). However, GOAT is also expressed in the brain, particularly in the hippocampus (Murtuza and Isokawa, 2018) which is also an area of abundant GHSR-1a mRNA (Zigman et al., 2006; Hornsby et al., 2020) and protein expression (Mani et al., 2014; Hornsby et al., 2016). The central expression of GOAT and GHSR-1a suggest that local acylation and subsequent receptor activation following the import of UAG may be possible (Murtuza and Isokawa, 2018). Notably, GOAT mRNA is reduced in brain samples from AD patients (Gahete et al., 2011) and is specifically reduced in the granule cell layer within the dentate gyrus (DG) of PD Dementia (PDD) patients compared to both PD and healthy control groups (Hornsby et al., 2020). These studies suggest that acylation of ghrelin is associated with dementia in humans.

## Acyl-Ghrelin Binding to GHS-R1a

**FIGURE 2:**
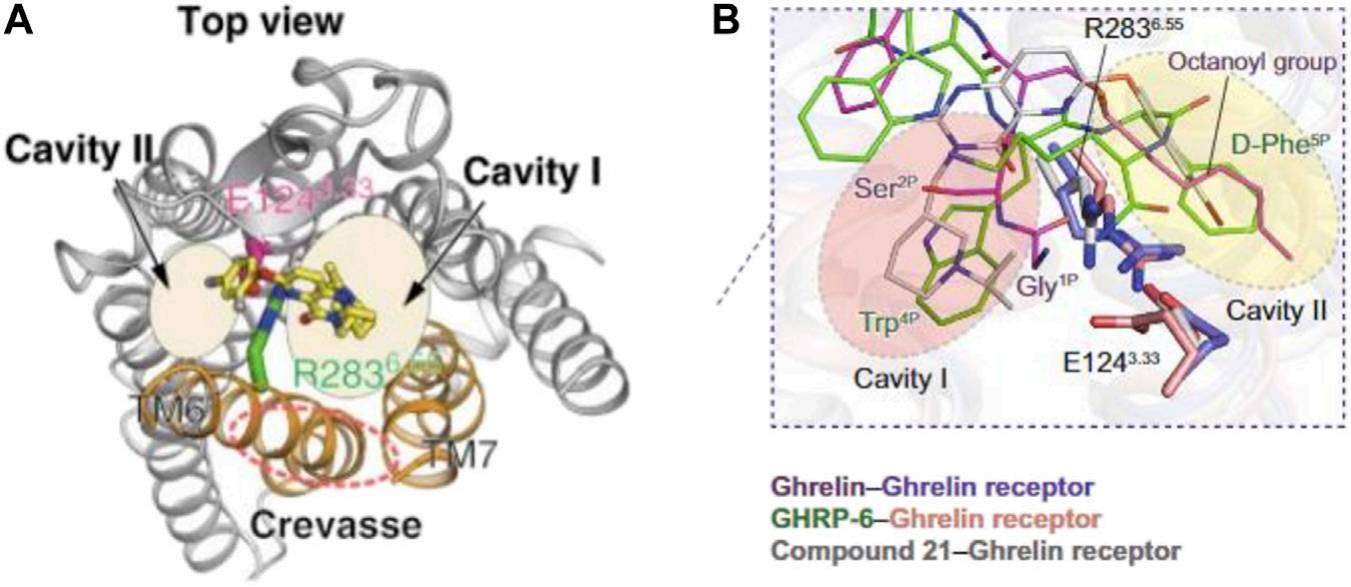
Structure modelling of the ghrelin receptor whilst bound to ghrelin. Structures proposed by Shiimura et al. (2020) **(A)** and Wang et al (2021) **(B)** both identify the ligand-binding pocket of ghrelin receptor being bifurcated into two cavities. The model proposed in **(A)** presents the octanoyl group inserted in a crevasse near cavity 1. Whilst the model proposed in **(B)** locates the octanoyl group within the second cavity.

Several experimental and *in-silico* strategies have been used to understand the rules for acyl-ghrelin binding and activating GHS-R1a. Liposome binding, zeta potential and isothermal calorimetry were used to show that acyl-ghrelin—but not UAG—penetrated the headgroup and partially into the lipid backbone regions of membranes (Staes et al., 2010). Importantly, acylation increased the concentration of the peptide in the membrane 120-fold, increasing its chances of engaging its receptor. Of note, several hydrophobic amino acids at the N-terminal—where the octanoyl moiety is attached—were shown to be critical for the close association of ghrelin with membranes. Consistent with this, a model of membrane-associated ghrelin inferred from Rosetta modelling based on NMR studies of the peptide in lipid vesicles suggest that ghrelin binds to membranes *via* the octanoyl moiety on Ser3 and with the contribution of basic amino acids electrostatically interacting with the lipid polar heads (Vortmeier et al., 2015). These amino acids were later characterized and the N-terminal binding motif of acyl-ghrelin was confirmed to substantially contribute to the binding energy of the peptide-receptor complex and ghrelin proposed to assume a rigid helical conformation involving C-terminal residues (Bender et al., 2019). This two-site binding mode where the octanoyl chain stabilizes the structure of the hormone and promotes its binding to the receptor was later confirmed (Ferré et al., 2019). Here, the utilization of solid-state NMR of lipid nanodiscs combined with coarse-grained molecular dynamic simulations substantially confirmed previous models (Bender et al., 2019) and experimental data (Bednarket et al., 2000; Matsumoto et al., 2001). The octanoyl chain strongly facilitates access of acyl-ghrelin to deeper binding pockets of the receptor as compared to UAG. Subsequently, acyl-ghrelin binds the receptor establishing a tight interaction with N-terminal residues, leading to the specific formation of a hydrophobic core, while the C-terminal region remains flexible. The hydrophobic core gives rise to the most stable peptide-receptor interactions which rigidifies the peptide in the bound state. However, the acyl chain seems not to interact with specific amino acids engaging in specific receptor-ligand interactions but rather effectively contributing to the formation of the hydrophobic core required to engage the deeper binding sites (Ferré et al., 2019). These data agree with previous studies showing that substitution of the C8 moiety with bulky hydrophobic amino acids are tolerated while polar or charged modifications hamper binding and activation of the receptor. As discussed, the preference for the octanoyl fatty acid likely resides in GOAT’s acyl-chain specificity (Darling et al., 2015). The crystal structure of the receptor bound to a neutral antagonist was resolved and in combination with mutagenesis and receptor binding and activity analyses, results showed that GHS-R1a shared similarities with both peptide hormone GPCRs and lipid GPCRs while possessing unique structural features. Particularly, the binding pocket of the receptor is uniquely crossed by a salt bridge—with mutations completely abolishing function—dividing it into two cavities. Mutations of polar amino acids in cavity 1 with hydrophobic alanine also abolish activity demonstrating their involvement in peptide recognition. However, similarly to lipid GPCRs, the ghrelin receptor also presents a wide gap (crevasse) which is enriched with hydrophobic phenylalanine residues, spacing between trans-membrane bundles 6 and 7. In the prototypical lipid GPCRs, cannabinoid-1 and sphingosine-1-phosphate receptors, similar phenylalanine residues recognize the lipoligands, whilst the ghrelin receptor accommodates the acyl moiety of ghrelin into this hydrophobic environment (Figure 2A) (Shiimura et al., 2020). These interactions allow for acyl-ghrelin recognition and are postulated to induce the transformation of the receptor into its active form according to a mechanism common to class A GPCRs (Zhou et al., 2019). Adding further complexity, cryo-electron microscopy structures of the ghrelin receptor in complex with an engineered G-protein and bound either to acyl-ghrelin or to a synthetic agonist show a bifurcated cavity with the N-terminus of acyl-ghrelin deeply inserted and occupying almost the entire binding pocket (Wang et al., 2021). Again, mutations of the amino acids involved in the salt bridge and of the hydrophobic amino acids in the binding pocket largely decreased acyl-ghrelin’s activity—supporting the critical role of these structures in binding and activating the receptor. However, different from previous modelling studies, here the octanoyl group is located at cavity 2 instead of being inserted in a crevasse near cavity 1. Additionally, the authors also propose that after binding in cavity 2, the octanoyl group orients the rigid N-terminus towards cavity 1 leading to initiation of signal transduction through conformational changes involving specific transmembrane bundles 6 and 7. Finally, several micro-switches or small conformational changes are induced to facilitate receptor-G protein coupling. The authors also postulate that hydrophobic interactions at the bundles above, together with the permanent salt bridge may be involved in basal activity, as this decreases severely upon mutation of the amino acids involved (Figure 2B) (Wang et al., 2021).

## *In Situ* Brain Acylation of Ghrelin and Crossing the Blood-Brain Barrier

**FIGURE 3:**
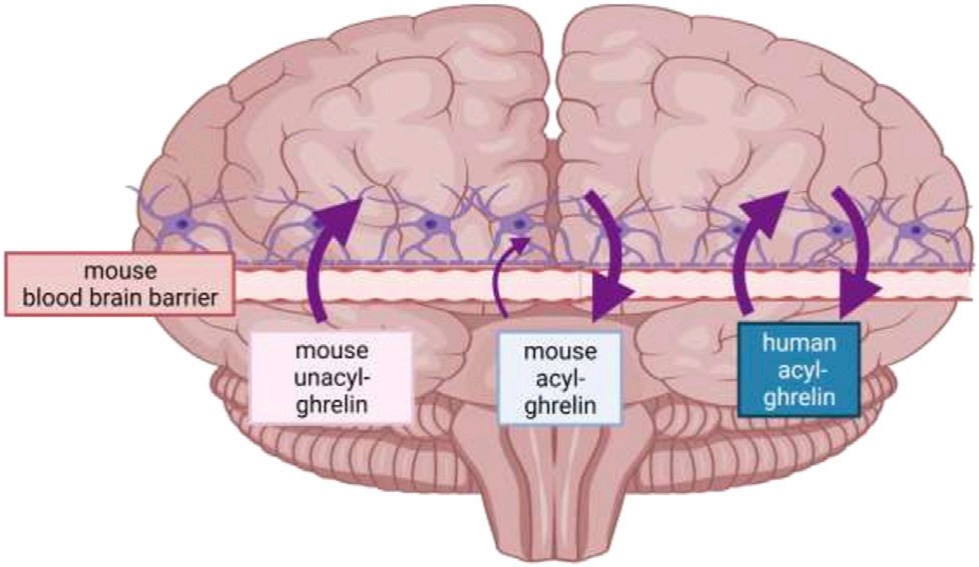
Direction of travel of human acyl-ghrelin, mouse acyl-ghrelin and mouse unacyl-ghrelin across the mouse BBB. Mouse acyl-ghrelin predominantly crosses the mouse BBB in the brain-to-blood direction, mouse unacyl-ghrelin is transported only in a blood-to-brain direction, whilst human acyl-ghrelin, which differs from mouse ghrelin at two amino acid residues is transported in both directions across the mouse BBB. As a result, ghrelin transport across the BBB is influenced by at least two features of its structure-its amino acid sequence and post-translational acylation (created with Biorender, adapted from Banks et al., 2002).

The brain is shielded by the blood-brain barrier (BBB) which selectively restricts access to the brain. Both acyl-ghrelin and UAG can be imported directly across the BBB from the periphery, with the acylation status reported to alter its ability to cross the BBB into the parenchyma (Figure 3) (Banks et al., 2002). Banks et al. demonstrated that radio-labelled human acyl-ghrelin crossed the mouse BBB *via* a saturable brain import and export system, whilst mouse acyl-ghrelin was only saturable during brain-to-blood transport. As only two of the 28 residues differ between mouse and human ghrelin these particular amino acids may be critical for recognition and transport from the blood to the brain, but not necessarily for brain-to-blood transport. The same study also reported that mouse UAG accessed the brain *via* non-saturable transmembrane diffusion, suggesting that it might move from the circulation into the brain for local acylation by GOAT (Murtuza and Isokawa, 2018). These initial findings were later supported by radiolabelled ghrelin studies suggesting that human acyl-ghrelin crosses the BBB *via* endocytosis (Diano et al., 2006; Pan et al., 2006). More recently, peripheral injections of F-ghrelin (a fluorescent analogue of acyl-ghrelin) resulted in its internalization by ependymal cells of the choroid plexus and by a specific subset of tanycytes, highly specialized ependymal cells that form a blood-cerebrospinal fluid (CSF) barrier (Uriarte et al., 2019). Whilst the exact mechanism of ghrelin’s entry into the brain is unknown, acyl-ghrelin’s accessibility seems to occur in a dose-dependent manner. Indeed, a low dose of a fluorescent acyl-ghrelin analogue injected subcutaneously in mice was scarcely present in the CSF and demonstrated a higher preference for the hypothalamus. While intracerebroventricular (icv) injected acyl-ghrelin (or high doses injected peripherally) was detected at high levels in the CSF and several brain regions (Cabral et al., 2013; Uriarte et al., 2019).

## Fatty Acid Substrates for Ghrelin Acylation

In humans and rats, acyl-ghrelin is predominantly bound by octanoic acid (C8:0, a saturated eight carbon fatty acid, also commonly known as Caprylic acid) (Kojima et al., 1999). Although this appears to be the physiologically relevant form, ghrelin may bind a range of saturated fatty acids that vary in length from 2 to 16 carbons (Hosoda et al., 2003; Nishi et al., 2005b; Gutierrez et al., 2008). The development of a cell-based system whereby fatty acids added exogenously to cells resulted in the formation of acyl-ghrelin with the corresponding fatty acid chain used to demonstrate that even non-endogenous fatty acids can be employed for the bio-acylation of ghrelin (Gutierrez et al., 2008; Gutierrez et al., 2008; Oiso et al., 2013). However, given that ghrelin acylation may be modulated by increased bioavailability of specific fatty acids the discussion regarding the preferred acyl donor group for ghrelin acylation by GOAT resides in the enzyme specificity. Indeed, several studies report that C8:0 is the preferred fatty acid substrate for acylation by GOAT, with its affinity being approximately 50-fold higher in comparison to C6:0 (hexanoic acid) and C10:0 (decanoic acid) (Kaiya et al., 2001; Darling et al., 2015) and 10:1 (decanoic acid, a monounsaturated fatty acid) (Hosoda et al., 2003). Conversely, Ohgusu et al. (2009) reported that GOAT prefers C6:0 as the acyl donor, followed by C8:0 then C10:0, and may not attach longer fatty acids such as C16:0 (palmitic acid) to ghrelin. Yang et al. (2008b) also suggest that GOAT may not be able to incorporate palmitate onto ghrelin. Notably, the attachment of C16:0 (palmitoylation) or C14:0 (myristic acid, myristylation) are the most common forms of acylation for a wide range of proteins (for a review please see Resh, 2016).

Distinct species of acyl-ghrelin differ in their efficiency to activate GHS-R1a (Bednarek et al., 2000; Date et al., 2000; Kaiya et al., 2001; Matsumoto et al., 2001; Hosoda et al., 2003; Nishi et al., 2005a; Gutierrez et al., 2008; Heppner et al., 2012). A fatty acid chain greater than two carbons was needed for activating GHS-R1a (Heppner et al., 2012). In addition, C2:0 (acetic acid), C8:0, C12:0 (dodecanoic acid), C14:0 and C16:0 forms of acyl-ghrelin had distinct effects on food intake and fat mass. Interestingly, C16:0-ghrelin led to a delayed (∼24 h) effect on stimulating feeding in comparison to the more immediate effect of C8:0-ghrelin (Heppner et al., 2012). Thus, studies to distinguish the acyl group added to ghrelin under both physiological and pathophysiological conditions are warranted. As part of this, one should consider the source of the acyl substrate and their respective metabolic pathways as targets to alter the profile of ghrelin species and subsequent GHS-R1a signalling.

There are several possible sources of fatty acids for the acylation of ghrelin, including, breakdown of longer-chain fatty acids (beta-oxidation), *de novo* fatty acid synthesis, and diet. Despite this the origin of the acyl moiety within the brain and stomach is still unknown. In addition, these processes may be modulated by the body’s microbiome. Here, we discuss putative biochemical mechanisms that may underpin both basal and appetite-associated acylation of ghrelin in the circulation.

## Beta Oxidation of Longer Chain Fatty Acids

Fatty acids are a major source of energy in animals and the main pathway for their biological degradation, namely β-oxidation, is present in both mitochondria (Kennedyt and Lehninger, 1949) and peroxisomes (Lazarow and De Duve, 1976). The two organelles regulate this pathway with both similarities and differences due to their enzymatic inventory. For this review, “very long-chain” is defined as having a number of carbons >20, “long-chain” comprised between 20 and 16, “medium-chain” between 14 and 8, and “short-chain” < 8. β-oxidation is the process where fatty acids are degraded in a stepwise manner to support energy production. As relevant to this review, MCFA such as octanoic acid used for ghrelin acylation, as well as SCFA, are water-soluble and may enter cells and organelles through unselective membrane pores (Papamandjaris et al., 1998; Antonenkov and Hiltunen, 2012).

**FIGURE 4:**
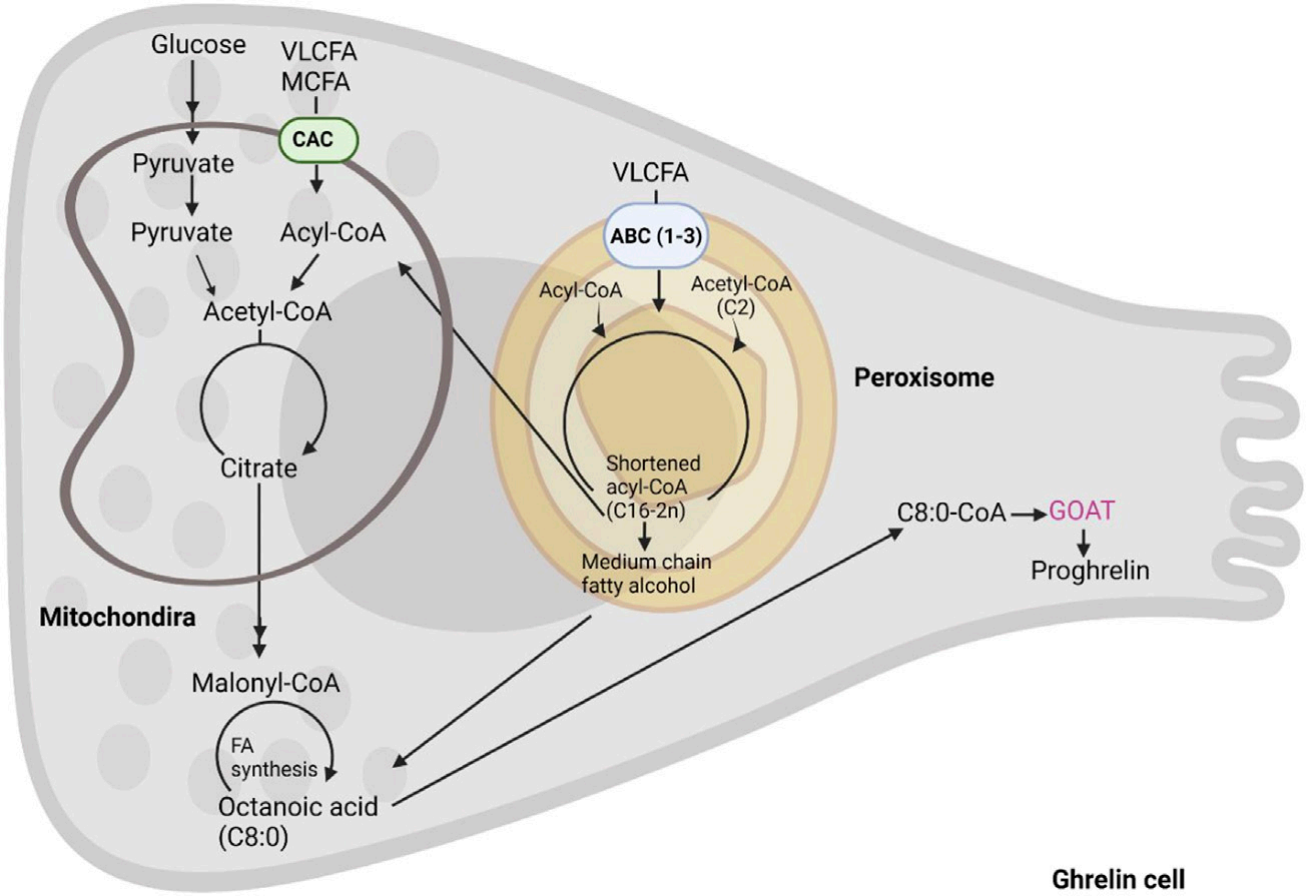
The formation of octanoic acid in the enteroendocrine cell. Acyl-ghrelin is predominantly bound by C8:0. In this schematic we summarize the intracellular C8:0 forming pathways including, fatty acids from diet, fatty acid synthesis and beta-oxidation of long chain fatty acids.

Conversely, hydrophobic LCFA and VLCFA utilize membrane transporters after being activated by esterification into acyl-CoA (Figure 4). The latter reaction is carried out by ACSL and FATP proteins whose differential localization within the two organelles is yet to be fully deciphered, for a review see Schrader et al. (2015). Also, the fatty acid transporters are different in mitochondria and peroxisomes where the former are equipped with the carnitine shuttle (CPT2, CACT, and CPT1) and the latter with three ABC transporters (ABCD1-3 (Figure 4)). Peroxisomes also possess a carnitine shuttle but whilst the mitochondrial transporter is used to import LCFA, the peroxisomal transporter exports MCFA. Indeed, after import, peroxisomes do not degrade fatty acids to completion, and according to the substrate affinities of their respective enzymes only shorten the chain length of fatty acids to approximately C8-C6. Intriguingly, C8-C6 are those preferentially used by GOAT for ghrelin acylation. These are exported from peroxisomes through a carnitine shuttle or free diffusion and then imported into mitochondria for further oxidation. In contrast to the mitochondrial carnitine shuttle, peroxisomal ABCD transporters prefer VLCFA (and dicarboxylic acids), thus the initial transporters function as filters trafficking fatty acids to the two organelles depending on their chain length. These findings demonstrate that both mitochondrial and peroxisomal beta-oxidation may play a role in supporting ghrelin acylation by providing the necessary fatty acyl moieties. However, studies where ghrelin levels have been measured with concomitant inhibition or depletion of specific mitochondrial and peroxisomal beta-oxidation enzymes is warranted.

Both acylated and unacylated ghrelin can stimulate fatty acid oxidation in skeletal muscle (Kraft et al., 2019) and myocytes (Han et al., 2015), and continuous administration of acyl-ghrelin enhanced muscle mitochondrial oxidative capacity in rats (Barazzoni et al., 2005; Guillory et al., 2017). Bolus ICV injections of ghrelin to rats led to an increase in long-chain acylcarnitines, which is indicative of increased beta-oxidation (Gao et al., 2013). Acyl-ghrelin administration increased the activity of carnitine palmitoyl transferase (CPT1), which transports fatty acyl-CoA into mitochondria for oxidation in the mouse hypothalamus (Andrews et al., 2008). Furthermore, in a transgenic mouse model where genes for both human ghrelin and GOAT are overexpressed, there was significantly decreased mRNA expression of Cytochrome C and uncoupling protein-3 (UCP-3) in the skeletal muscle (Kirchner et al., 2009). These results are indicative of increased beta-oxidation in the mitochondria (Schrauwen et al., 2001; Andrews et al., 2005; Andrews et al., 2009; Andrews et al., 2010).

Several studies have investigated whether fatty acids longer than C8:0, particularly those introduced *via* the diet, can increase acyl-ghrelin. ST-Onge et al. (2014) showed that both MCT and LCT increased levels of acyl-ghrelin in overweight humans (St-Onge et al., 2014). Whilst intravenous infusion of LCFA increased both acyl-ghrelin and the AG: TG ratio in rats (Barazzoni et al., 2017). Could essential omega 3 fatty acids represent precursors for acylating ghrelin? Several studies link these particular fatty acids and ghrelin. Indeed, a fatty seafood diet, rich in DHA and EPA, increased ghrelin in the blood of humans (Ramel et al., 2009). Furthermore, omega-3 supplementation elevated serum ghrelin levels in both rats (Mostafa et al., 2020) and humans (Rahimi and Khademi, 2019). High-capacity runner rats fed a balanced diet (containing ×10 more fish oil, and therefore more omega 3 fatty acids) had higher plasma levels of acylated ghrelin in comparison to those on an unbalanced diet (Ramel et al., 2009). Furthermore, ghrelin levels in rat plasma were significantly higher after a high-fat high-sucrose diet supplemented with both DHA and EPA (1:1) and grape seed extract (Ramos-Romero et al., 2016). Consistent with these studies, LCFA was needed for the acylation of ghrelin in a ghrelinoma cell line (MGN3-1) (Bando et al., 2016).

Conversely, the oxidation of longer fatty acids may not be required for ghrelin acylation. For example, a 12-week dietary intervention where humans ingested either commercial goat cheese or goat cheese enriched with omega-3 PUFAs and linoleic acid did not lead to altered ghrelin levels (Santurion et al., 2020). Another study in humans compared low and high omega-3 dietary regimes for 6 weeks also showed no difference in serum ghrelin (Parra et al., 2008). More specifically, oleic acid (18:1) increased preproghrelin mRNA expression when cultured with intestines and hepatopancreas cells from goldfish (Bertucci et al., 2017). However, no effect was seen in response to linoleic acid (18:2) or EPA, whilst DHA led to a decrease in preproghrelin mRNA and protein (Bertucci et al., 2017). Furthermore, Haynes et al. (2020) reported that effects induced by octanoic acid on pro-opiomelanocortin (POMC) neurones, such as ion channel opening, were not subsequently seen by the LCFAs oleic and palmitic acids (Haynes et al., 2020). It seems that rather than being potential precursors for the acyl modification of ghrelin, some LCFA or VLCFA act to decrease acyl-ghrelin. Indeed, gastric gavage of LCFAs significantly decreased ghrelin secretion in the serum of mice (Lu et al., 2012). Also, the culture of ghrelin expressing gastric cells with docosadienoic acid (C22:2), linoleic acid (18:2) and palmitic acid (16:0) reduced ghrelin secretion (Lu et al., 2012). Oiso et al. (2015) investigated both the *in vitro* and an *in vivo* effect of different fatty acids (acetic acid (C2:0), stearic acid (18:0), oleic acid (18:1), linoleic acid (18:2) and α-linoleic acid (18:3)) on octanoylated ghrelin in AGS-GHRL8 cells (a gastric carcinoma cell line that expresses ghrelin) (Oiso et al., 2015). They reported that stearic, oleic, linoleic and α-linoleic acids significantly reduced the amount of octanoyl-ghrelin secreted into the culture medium. Similarly, the administration of ethyl oleate (which can be converted to oleic acid in the body) to mice significantly reduced both octanoylated- and unacyl-ghrelin in serum (Oiso et al., 2015).

The ghrelinergic cell line (MGN3-1) α-linoleic acid led to a concentration-dependent decrease in octanoyl-ghrelin that seemed to be modulated by gustducin (Janssen et al., 2012). Conversely, the clearance of ghrelin may be mediated by the fatty acid receptor, GPR120 (Widmayer et al., 2017). A GPR120 agonist GW9508 significantly decreased the secretion of acyl-ghrelin, which was rescued after treatment with GPR120 siRNA. A fasting-induced increase in plasma acyl-ghrelin was also blocked in GW9508 treated mice (Gong et al., 2014).

In summary, several studies indicate that food consumption acts as an endogenous source of MCFA, including octanoic acid, for ghrelin acylation. However, it should be noted that in these studies the levels of acyl-ghrelin are the result of an equilibrium between acylation by GOAT and de-esterification by ATP1 or similar esterases. At present, and to the best of our knowledge, the effect on acyl-ghrelin following the use of fatty acid β-oxidation inhibitors has not been delineated in animals. However, should their findings be confirmed *in vivo* they may imply that beta-oxidation is the major source of octanoic for ghrelin acylation, considering that the same study also explored the potential impact of the intestinal microbiome and fatty acid synthesis onto the production of MCFAs for ghrelin acylation but did not find data in support. Fatty acids synthesis, the diet and the microbiome can, however, modulate ghrelin acylation and in a specific setting and are therefore reviewed in the following. In summarizing the findings above we may propose a feedback mechanism linking ghrelin and beta-oxidation. Inhibition of β-oxidation (with mitochondrial CPT1 inhibitors) decreased ghrelin acylation *in vitro* and many studies above have shown that such inhibition also decreased its orexigenic effects. Therefore, beta-oxidation would be required both for ghrelin acylation and to mediate its effects (or at least some of them). Ghrelin also interacts with PPAR-γ which induces peroxisomal and mitochondrial biogenesis while interactions with PPAR-α or -δ, which can induce expression of beta-oxidation enzymes, are not known. These data suggest that ghrelin is stimulating β-oxidation (or at least preparing cells for that purpose) when substrate is low (e.g., during starvation) to prevent hypoglycaemia and death. Given that more data is needed to conclude beta-oxidation donating the C8 for acylation, we speculate that during periods of fasting/hunger beta-oxidation of adipose tissue sustains ghrelin acylation and in turn acyl-ghrelin positively upregulates beta-oxidation.

## Fatty Acids From the Diet

Analyses of acyl-ghrelin and medium-chain fatty acids (MCFAs) in human plasma during fasting report no correlation, suggesting that fatty acids for ghrelin acylation may preferentially derive from gastric content rather than plasma (Nass et al., 2015). The diet is a rich source of MCFA, with dairy products such as milk and oils from palm kernel and coconuts enriched in MCFAs such as octanoic acid (Jensen et al., 1990; Takeuchi et al., 2008). Ingested MCFA and medium-chain triacylglycerols (MCTs) (a glycerol backbone attached to three fatty acids) elevated levels of acyl-ghrelin within the stomach without inducing the expression of total ghrelin mRNA or peptide (Nishi et al., 2005b). More specifically, ingestion of either MCTs including glyceryl trihexanoate (3xC6:0), glyceryl tripheptanoate (3xC7:0), glyceryl trioctanoate (3xC8:0) or glyceryl tridecanoate (3xC10:0) or the corresponding MCFAs (hexanoic, octanoic or decanoic acids) by mice, increased the acyl-ghrelin species in the stomach containing the corresponding number of carbons i.e., hexanoyl-ghrelin, heptanoyl-ghrelin, octanoyl-ghrelin or decanoyl-ghrelin, respectively. However, this effect was not seen in mice fed the glyceryl tributyrate (C4:0), glyceryl trilaurate (C12:0) or glyceryl tripalmitate (C16:0) suggesting that fatty acids from C6 to C8 are preferred by GOAT. A subsequent study in 2005, showed that rats fed with chow containing glyceryl trioctanoate led to the detection of octanoyl-ghrelin in their gastric content (Nishi et al., 2005a). Mice fed with a MCT diet of triheptanoate, trioctanoate or tridecanoate led directly to increased levels of heptanoyl, octanoyl- or decanoyl-ghrelin, respectively, in the plasma (Kirchner et al., 2009). This study also included transgenic mice which overexpress both the human ghrelin (GHRL) and GOAT (MBOAT4) genes in the liver. These mice did not produce octanoylated human ghrelin when fed regular chow (only octanoylated mouse ghrelin), however, feeding with trioctanoate led to the presence of circulating octanoylated human ghrelin (Kirchner et al., 2009). Several other studies provide further support for diets enriched with octanoic acid increasing plasma acyl-ghrelin levels, including in plasma of pigs (Miller et al., 2016) and cachectic patients undergoing enteral feeding (Ashitani et al., 2009), demonstrating that acyl-ghrelin levels can be modulated by feeding. The same effect was reported in the stomachs of neonatal chickens (Yamato et al., 2005) and plasma, liver and stomach of suckling rats (Lemarié et al., 2016). Furthermore, a diet enriched with MCTs increased levels of acyl-ghrelin in overweight humans where acyl-ghrelin levels are low (Yamato et al., 2005). Fukumori et al. (2013) also showed that dairy cows fed a diet with increased content of C8:0, C10:0 and C12:0 led to increased plasma total ghrelin (TG) (Fukumori et al., 2013). Of particular interest, a diet enriched with octanoate did not lead to an increase in plasma acyl-ghrelin in rats, but rather led to a decrease in UAG, resulting in an increased AG:TG ratio. Acyl-ghrelin detected in gastric cells correlated with the dose of dietary tioctanoin, which supports the theory of direct absorption of the fatty acyl substrate for ghrelin acylation *via* the gut (Lemarié et al., 2015).

Conversely, Kaur et al. (2020) showed that the addition of glyceryl trioctanoate to the diet of pregnant mice increased total ghrelin but not acyl-ghrelin (Kaur et al., 2020). However, this study did not compare the levels of acyl-ghrelin detected in mice on glyceryl trioctanoate to mice on standard chow, but rather, to mice on a diet enriched with glyceryl tripalmitate (C16:0). Their rationale being that dietary palmitate does not lead to ghrelin acylation (Nishi et al., 2005b). However, as C16:0 can bind ghrelin (Gutierrez et al., 2008) or even be a precursor for octanoic acid (*via* beta-oxidation), these results should be interpreted carefully. Most of the studies described above suggest that dietary-derived MCFAs or MCTs can be used for ghrelin acylation in the gut.

Indeed, GOAT may act as a gastric lipid sensor, linking ingested nutrients with the hypothalamic energy balance regulation *via* the ghrelin system. More specifically, the activation of GOAT in the gut is suggested to link to the availability of the lipid pool, or more specifically to the MCFA content, as MBOAT4 mRNA expression is downregulated in the absence of MCFA (Kirchner et al., 2009). However, a diet with increased glyceryl octanoate did not affect the mRNA expression of ghrelin or MBOAT4 in pregnant mice (Kaur et al., 2020). There is also evidence that UAG may promote the uptake of fatty acids by cells for acylation. Indeed, using a fluorescent form of C12:0, UAG increased the uptake of this fatty acid into cardiomyocytes (Lear et al., 2010). In summary, dietary MCT may directly influence the ghrelin species present in the stomach, however, further investigation of other tissues is required in this context.

## Fatty Acid Synthesis

During dietary insufficiency, the brain (Knobloch et al., 2013), liver (Dorn et al., 2010) and adipose tissues (Mayas et al., 2010) can synthesize fatty acids (Skulachev 1991; Menendez and Lupu 2007; Knobloch et al., 2013). During this process, fatty acid synthase (FAS) can synthesize longer chain fatty acids (Maier et al., 2006) (Figure 4). However, MCFA synthesis has only been reported in goat mammary glands (Knudsen and Grunnet, 1982). More specifically, the synthesis of octanoic acid has been described in the lactating glands of rats (Libertini and Smiths, 1978) and octanoylated ghrelin has also been detected in the lactating mammary glands of both dairy goats (Zhang et al., 2018) and humans (Grönberg et al., 2010). However, several studies suggest that the fatty acid for ghrelin modification is not predominantly derived from fatty acid synthesis. For example, Lopez et al. (2008) reported that fasting and ghrelin treatment led to a decrease in FAS in the rat hypothalamus, which is regulated by the metabolic sensor, AMPK. Acyl-ghrelin treatment of rats with a bolus intracerebroventricular (ICV) injection led to a decrease in Malonyl-CoA and long-chain acyl-CoA in their ventral medial nucleus, which are indicative of decreased fatty acid synthesis (Gao et al., 2013). Furthermore, neither acylated nor UAG altered the incorporation of fatty acids into TAGs or phospholipids (Kraft et al., 2019). AMPK-mediated phosphorylation of acetyl-CoA carboxylase (ACC)-1 inhibits fatty acid synthesis (Lopez et al., 2008; Peterson et al., 2012; Adams, 2004), whilst phosphorylating ACC-2 promotes beta-oxidation (O'Neill et al., 2014; Fullerton et al., 2013). Notably, mRNA expression of the SCFA receptor, GPR43, was higher in gastric-derived ghrelin positive cells in comparison to ghrelin negative cells. However, incubation of these with butyric acid (C4:0) or valeric acid (C5:0) did not affect ghrelin secretion (Lu et al., 2012). The SCFA, acetate (C2:0), reduced appetite after crossing the BBB (Frost et al., 2014), suggesting that it could act as a competitive inhibitor of octanoyl-ghrelin. Further work is required to determine whether fatty acid synthesis and ghrelin acylation are directly linked, particularly in the context of cell-type specific ghrelin action.

## Fatty Acids From the Microbiome

The co-habitant bacteria in the gut, or the microbiome, is also a source of fatty acids that could be used to acylate ghrelin. The most abundant gut microbiota-derived metabolites include the SCFAs acetate (C2:0), propionate (C3:0) and butyrate (C4:0) (Topping and Clifton, 2001), which are synthesized during the fermentation process of nutrients in the colon. There are also reports of the microbiome utilizing diet-derived MCFAs and LCFAs (Zhao et al., 2018; Radka et al., 2020). For example, MCFAs and LCFAs have been shown to promote and inhibit different types of pathogenic or symbiotic bacteria, respectively, in addition to having antibacterial properties (Fischer et al., 2012; van der Hoeven-Hangoor et al., 2013). The gut microbiome has been associated with ghrelin function. Generally, the microbiome plays a role in numerous activities which overlap with the function of GHSR-1a, including obesity (Torres-Fuentes et al., 2017), adult hippocampal neurogenesis (Ogbonnaya et al., 2015) and metabolism (Cox et al., 2014). More directly, acetate derived from gut microbiota can stimulate ghrelin secretion (Perry et al., 2016), whilst circulating levels of ghrelin are seemingly linked to the microbiome. For example, ghrelin levels were negatively correlated with *Lactobacillus, Bifidobacterium*, and *B. coccoides Eubacterium rectale*, but positively correlated with *Bacteroides* and *Prevotella* (Queipo-Ortuño et al., 2013)*.* Interestingly, germ-free mice have decreased ghrelin in their blood (Duca et al., 2012). Also, a probiotic diet in chickens led to an increase in ghrelin gene expression in their proventriculus (the glandular section of their digestive system) (Poorghasemi et al., 2018). Also, the culture of primary stomach cells from mice with *Lactobacillus brevis* SBC8803 resulted in an increase in acyl-ghrelin in the supernatant (Saito et al., 2019). Importantly, the ratio of AG:UAG was also significantly increased in rats 30 min after oral administration of *Lactobacillus brevis* SBC8803 (Saito et al., 2019). Notably, the transcription of ghrelin or GOAT mRNA was unchanged, suggesting that the increase may be due to promoting the acylation of ghrelin. Furthermore, bacteria-derived SCFAs (Cherbut et al., 1997; Dass et al., 2007) and LCFAs (Zhao et al., 2018) have been linked to increased gut motility, a function of acyl-ghrelin signaling (Fujimiya et al., 2012). However, a recent report suggested that the microbiome did not modulate plasma acyl-ghrelin in mice (Ikenoya et al, 2018). Whether the gut microbiome can regulate brain function *via* the acylation of ghrelin has yet to be fully investigated.

## Fatty Acids Into the Brain

The ability of fatty acids to traverse the BBB or the blood-CSF barrier should be considered. Tight junctions formed in the BBB mean that free fatty acids enter the brain either *via* passive diffusion or they are assisted by proteins, however, the exact mechanism for fatty acid import is still unclear. Fatty acid diffusion depends on the length of the fatty acid chain which can impact their solubility (Kamp et al., 2003; For a review see Hamilton, 1998). SCFAs and MCFA’s traverse the BBB by diffusion whilst transport of longer chain fatty acids is slower and requires assistance, for example, by the fatty acid transport protein (FATP)-1 (Kamp et al., 2003). Both essential fatty acids, such as linoleic acid (18:2) (DeMar et al., 2006) and non-essential fatty acids e.g., arachidonic acid (20:4) and palmitic acid (16:0) are derived from the blood (DeMar et al., 2006). Importantly, octanoic acid enters the brain *via* diffusion, shown *in vitro* (human U373-MG GBM cells (Yamazaki et al., 1996), in brain slice preparations (Kuge et al., 2002) and in rats (Yamazaki et al., 1996), dogs (Kuge et al., 2000) and cats (Kuge et al., 2000), where the ability of radiolabelled octanoic acid to enter the brain was quantified using brain PET imaging. Furthermore, octanoic acid administered directly into the CSF of mice was rapidly transported into the hypothalamus *via* GPR40 (Haynes et al., 2020). Radiolabelled octanoic acid administered into the mouse gut by oral gavage also entered the hypothalamus, whilst an oral gavage of the MCT octanoate decreased food intake and increased melanocortin secretion from the hypothalamus, a hormone that is linked to regulation of energy homeostasis (Haynes et al., 2020). In the same study, octanoic acid readily crossed the BBB whilst LCFA (oleic and palmitic acids) transport was minimal. This is supported by radiolabelled dietary palmitic (16:0) or oleic acid (18:1) fed to rat pups remaining undetected in the brain, whilst other polyunsaturated fatty acids such as linoleic acid were detected in the brain (Edmond et al., 1998; Williams et al., 1997). However, C14 labelled oleic acid was reported to transport across confluent cultures of primary human brain microvessel endothelial cells (HBMEC) (Mitchell et al., 2009). Notably, essential fatty acids such as DHA and EPA cannot be synthesized by the body and must be derived from the diet. Thus, the fatty acid for ghrelin acylation in the brain may either be derived directly from the periphery or longer chain fatty acids that may be selectively imported across the BBB.

Collectively, the studies discussed in this review increase our understanding of ghrelin acylation and the underlying biochemistry. Further work unpicking the cell-specific mechanisms governing the acylation of ghrelin will likely yield novel insights into the integration of energy balance with brain plasticity linked to hippocampal neurogenesis and cognition.

## Glossary

ACC: Acetyl-CoA carboxylase
AD: Alzheimer’s disease
AEBSF: 4-(2-Aminoethyl)benzensulfonyl fluoride hydrochloride
AG: Acyl-ghrelin
AHN: adult hippocampal neurogenesis
APT: Acyl-protein thioesterase
BBB: blood brain barrier
BChE: butyrylcholinesterase
BDNF: brain derived neurotrophic factor
CNS: central nervous system
CR: calorie restriction
DG: dentate gyrus
DHA: docosahexaenoic acid
EPA: eicosapentaenoic acid
ER: endoplasmic reticulum
FAS: fatty acid synthase
FATP: fatty acid transport protein
FFA: free fatty acid
FFARs: free fatty acid receptors
GHRL: ghrelin
GHS-R1a: growth hormone secretagogue receptor-1a
MBOAT4: membrane bound O-acyl transferase
GOAT: ghrelin-o-acyltransferase
GPCR: G-protein coupled receptor
Hhat: hedgehog acyl transferase
LC-MS: liquid chromatography-mass spectrometry
LCT: long-chain triglycerides
MAFP: Methoxy arachidonyl fluprophosphate
MALDI-TOF: Matrix-Assisted Laser Desorption/Ionization-Time of Flight
MCFA: medium chain fatty acids
MCT: medium chain triacylglycerols
MS: mass spectrometry
NSPC: neural stem and progenitor cells
PAF: platelet activating factor
PC1/3: pro-hormone convertase 1/3
PD: Parkinson disease
PDD: Parkinson disease dementia
PUFA: polyunsaturated fatty acid
SCFA: short-chain fatty acid
SGZ: sub granular zone
UAG: unacyl-ghrelin
UCP: uncoupling protein
VLCFA: very long-chain fatty acid
